# Prevalence and Predictors of Digital Sexual Harassment Perpetration Among Youth: The Role of Demographics, Sexism, Moral Disengagement, and Online Ethical Values

**DOI:** 10.3390/bs15121642

**Published:** 2025-11-28

**Authors:** Mariana Alonso-Fernández, Jone Martínez-Bacaicoa, Marcos Romero-Suárez, Estíbaliz Mateos-Pérez, Manuel Gámez-Guadix

**Affiliations:** 1 Biological and Health Psychology Department, Autonomous University of Madrid, 28049 Madrid, Spain; mariana.alonso@uam.es (M.A.-F.); jone.martinez@uam.es (J.M.-B.); marcos.romero@uam.es (M.R.-S.); 2 Department of Social Psychology and Methodology of Behavior Science, University of Basque Country, 01006 Vitoria, Spain; estibaliz.mateos@ehu.eus

**Keywords:** technology-facilitated sexual violence, digital sexual harassment, moral disengagement, sexism, ethical values, youth

## Abstract

Digital sexual harassment (DSH) perpetration among youth is a concerning issue that requires further research attention. This study examined the prevalence of DSH perpetration according to gender, age, sexual orientation, and relationship status, and explored risk factors (hostile sexism, benevolent sexism, and moral disengagement) and protective factors (online ethical values). A total of 1098 Spanish adolescents and young adults aged 13–23 years (Mage = 16.07, SDage = 2.38) completed a self-report survey. Descriptive, correlational, and binomial regression analyses were conducted. Results showed that 13.4% of participants engaged in DSH in the past 12 months. Male participants reported more than twice the rates observed among female participants (21.1% vs. 7.9%), and adolescents reported higher prevalence than young adults, whereas no differences emerged for sexual orientation or relationship status. Regression analyses indicated that benevolent sexism was a consistent predictor, while gender moderated the effects of hostile sexism and moral disengagement. Hostile sexism predicted perpetration only among female participants and predicted moral disengagement only among male participants. Importantly, online ethical values emerged as a novel protective factor, substantially reducing the likelihood of perpetration and buffering, though not eliminating, the risks associated with high moral disengagement. These findings provide evidence for prevention strategies and underscore the role of ethical values in addressing gendered forms of online violence.

## 1. Introduction

The digitalization of societies in recent decades has driven progress across many areas, transforming how people communicate and form relationships. Increasingly, younger generations engage daily with digital devices and social networks, using them as their main means of communication and exploration of intimacy (Mateos-Pérez et al., 2025). Although online interaction can foster personal and social development by connecting people with supportive communities (Hiebert & Kortes-Miller, 2023), it also entails risks, particularly for adolescents who, feeling comfortable in online spaces, tend to underestimate the consequences of certain risky patterns of interaction (Donoso-Vázquez et al., 2018). Consequently, digital environments not only create opportunities for positive interaction but have also expanded the reach of various forms of structural violence by enabling them to take root in online contexts (Bailey et al., 2021; Patel & Roesch, 2022). A clear example of this is sexual violence, the digital expression of which continues to grow. Technology-Facilitated Sexual Violence (TFSV) refers to different forms of sexual abuse carried out through digital devices (Henry & Powell, 2018), including the non-consensual sharing of intimate images, threats to share sexual content (photos/videos), and digital sexual harassment (DSH), which includes various types of unwanted sexual attention behaviors, such as, for example, making improper sexual requests or sending unsolicited intimate images (Powell & Henry, 2019).

Over the last decade, many studies have focused on TFSV, showing that it is a widespread problem that can seriously impact the mental health of those who experience it (Champion et al., 2022). Despite this growing body of work, significant gaps remain in the literature. These are particularly evident in the case of DSH, one of the most prevalent expressions of TFSV (Gámez-Guadix et al., 2023; Martínez-Bacaicoa et al., 2024a), yet also one of the least explored, with most studies focusing primarily on victimization. In this regard, several studies have documented alarming rates of DSH victimization, ranging from 29% in the general population of Australian adults (Powell & Henry, 2019) to up to 84% among Canadian undergraduate women (Salerno-Ferraro et al., 2022). In Spain, recent data reported by Martínez-Bacaicoa et al. (2024b) provide a first-hand insight into the prevalence of DSH victimization, indicating that 74.2% of young adults have experienced some form of it, while Gámez-Guadix and Íncera (2021) found that 45.2% of non-heterosexual adolescents had experienced DSH. However, studies exploring direct perpetration experiences remain scarce and underdeveloped. While it is important to give visibility to victims, overlooking those who engage in violent behavior limits our ability to identify its causes and address the issue at its root.

Only a few European studies have specifically focused on the perpetration of DSH during adolescence (Franceschi et al., 2024), with recent Spanish data revealing that 34.5% of adolescents reported engaging in some form of DSH behavior (Durán-Guerrero et al., 2025). Despite these advances, further research is needed to deepen the understanding of the factors influencing DSH perpetration among youth. This is particularly relevant in Spain and other Mediterranean countries, where traditional gender norms that may support and legitimize sexist or aggressive peer behavior (Leaper & Brown, 2018) coexist with increasing digitalization and online interaction. Therefore, this study focuses on DSH perpetration among adolescents and young adults, a population in which TFSV has been found to be more prevalent (Miguel-Álvaro et al., 2025; Powell & Henry, 2019).

Specifically, this study examines the prevalence rates of perpetration, the role of sociodemographic characteristics, as well as both risk and protective factors. Among the risk factors, this study analyzes ambivalent sexism and moral disengagement—two constructs that have been widely studied as explanatory mechanisms of various forms of offline and online violence, including their recent application to TFSV (Durán-Guerrero & Sánchez-Jiménez, 2025; Martínez-Bacaicoa et al., 2024c). Sexism refers to a belief system that assumes gender norms—such as the idea that men have stronger sexual drives or that women are responsible for satisfying them—as natural truths (Leaper & Robnett, 2018). In addition, moral disengagement refers to a set of cognitive mechanisms that allow individuals to disconnect from internal moral standards, enabling them to engage in harmful behavior without experiencing guilt or self-condemnation (Bandura, 1986). However, their specific role in DSH perpetration has received limited attention in empirical research to date. Conversely, online ethical values, understood as personal principles that guide appropriate, responsible, and respectful behavior in digital environments (Wang et al., 2022), are analyzed as potential protective factors against the perpetration of DSH. Despite their relevance and previously demonstrated moderating role in online forms of aggression such as cyberbullying (Park & Bae, 2025), they remain an underexplored construct, especially in relation to gender-based forms of violence such as DSH. This approach seeks to contribute to a broader understanding of the factors that may promote or inhibit the perpetration of DSH. The following sections present key findings from the literature that help contextualize and guide this line of research.

### 1.1. Sociodemographic Variables Associated with Perpetration

Understanding the sociodemographic characteristics of those who perpetrate violence makes it possible to identify patterns and design prevention strategies tailored to the profiles most likely to be involved. In this regard, research not only points to females as the primary victims of DSH but also identifies males as the main perpetrators (Martínez-Bacaicoa et al., 2024d; Salazar et al., 2023; Snaychuk & O’Neill, 2020). These gender differences in experiences of both victimization and perpetration of DSH can be explained by the existence of social norms and expectations that portray men as inherently driven by sexual desire (Khera et al., 2022), and women as responsible for satisfying it (Mikorski & Szymanski, 2017). However, taking these into account does not mean assuming that gender identity automatically determines who is the aggressor and who is the victim. In fact, some studies have found that female participants can also perpetrate different forms of DSH, and that male participants can be victims as well (Erentzen et al., 2023). For example, Karasavva et al. (2023) found that some females sent unsolicited sexual images, often motivated by partner hunting, wanting to show off a body they felt proud of, or pursuing sexual gratification. According to the authors, these behaviors should be understood within a culture that reduces women’s value to their physical appearance—often prompting them to adopt a masculine gaze and to self-objectify (Chen et al., 2023)—and fosters the belief that any man would feel flattered to receive sexual content, even if unsolicited. In other words, treating certain male desires as “natural” can influence some heterosexual women to engage in behaviors involving unwanted sexual attention as a way of aligning with those expectations and enhancing their chances of finding a partner. However, although perpetration experiences may be shaped by gender, their differences have not yet been systematically explored.

Another population particularly affected by DSH is adolescents and young adults, not only due to their higher exposure rates, but also because the consequences of victimization at this age tend to be more negative (Martínez-Bacaicoa et al., 2024b). While research has mainly focused on victimization, perpetration rates among youth are also concerning, ranging from 7.6% in a U.S. sample to 48.5% in Croatia (Ramljak et al., 2025; Ybarra & Petras, 2021). The time spent on social media, combined with sensation seeking and risk-taking tendencies, may increase the likelihood of young people accessing certain internet applications and content—such as pornography—through which they may become more prone to perpetrating DSH (Stanley et al., 2018). Moreover, DSH may be perceived by adolescents as normalized or even expected within certain peer groups, especially among boys, but also among girls who internalize sexist beliefs and legitimize such behaviors (DeKeseredy et al., 2019). Therefore, it is essential to examine DSH perpetration among youth by considering the specific psychological and social characteristics of this developmental stage.

Another population group that has been found to be more affected by TFSV, including DSH, is lesbian, gay, and bisexual (LGB) people (Gámez-Guadix & Íncera, 2021; Powell et al., 2018). Certain stressors related to the social position attributed to LGB individuals—such as stigma, discrimination, or rejection—expose them to higher levels of both offline and online victimization (Amadori & Brighi, 2025). These homophobic attitudes are often amplified by the specific characteristics of online communication, such as perceived anonymity and the lack of physical contact, which can facilitate the perpetration of such forms of violence (Wright & Wachs, 2021). However, these same online features may also lead non-heterosexual youth to perceive certain digital spaces as safer environments in which to explore their sexual identity and connect with others to share experiences and build community (Hiebert & Kortes-Miller, 2023; Smith, 2022). These intersecting dynamics highlight the importance of investigating whether such factors also influence the likelihood of LGB youth engaging in DSH behaviors, an issue that remains largely underexplored in TFSV research (Patel & Roesch, 2022).

Finally, focusing on the relationship between the perpetrator and the recipient of DSH offers a valuable perspective, as relational norms—those that guide interactions within romantic relationships, friendships, or encounters with strangers—can significantly shape both the meaning of these behaviors and how victims respond to them (Hertzog & Rowley, 2014). For example, DSH may be directed not only at someone the perpetrator is attempting to pursue romantically, but also at current or former partners, friends, or even strangers met online (Salazar et al., 2023). Some studies have found that teenagers tend to reject unwanted sexual attention from strangers, but may struggle to say no when those messages come from a partner or someone they are close to (Lunde & Joleby, 2023). This dynamic may also suggest that some individuals feel entitled to engage in DSH depending on the type or closeness of the relationship. However, there is still a notable lack of research addressing this aspect, with most studies failing to specify the nature of the aggressor-victim relationship, as noted by Franceschi et al. (2024) in their recent scoping review.

Taken together, these insights highlight the importance of considering not only gender when analyzing the perpetration of DSH, but also other factors such as age, sexual orientation or the relational context in which it occurs, and how these shape the dynamics and meanings of these behaviors.

### 1.2. Risk Factors Contributing to Perpetration

According to the Ambivalent Sexism Theory (Glick & Fiske, 1996), sexism manifests in two forms: hostile sexism and benevolent sexism. Hostile sexism involves overtly negative attitudes toward women, including the belief that they seek to manipulate or dominate men. In contrast, benevolent sexism consists of seemingly positive but patronizing beliefs that portray women as pure, nurturing, and in need of male protection. Both forms of sexism have been recently found to be more common among male adolescents, who are also more likely to comment on girls’ bodies (Rodríguez-Castro et al., 2025). Similarly, sexist ideology has been linked to a higher likelihood of engaging in various forms of TFSV, such as non-consensual intimate image sharing (Morelli et al., 2016; Pina et al., 2017), cyberdating abuse in young couples (Ramírez-Carrasco et al., 2023) and online harassment in online video games (Tang et al., 2019). One explanation for these findings is that sexist ideology serves to justify sexual aggression by blaming women for “deserving it”, especially when they deviate from traditional gender stereotype regarding how a woman “should” be (Thompson, 2018), as well as within certain affective relational contexts among youth, where controlling behaviors are perceived as expressions of love and care (Sánchez-Hernández et al., 2020), thereby facilitating perpetration.

Another widely studied factor in explaining the justification of different forms of violence is moral disengagement, as extensive research has identified it as a key mechanism involved in adolescents’ perpetration of violent acts such as bullying (e.g., Gini et al., 2014; Killer et al., 2019), cyberbullying (e.g., Falla et al., 2023; Paciello et al., 2020), and online hate speech (e.g., Wachs et al., 2022). In the context of sexual violence, prior studies have shown that adolescent boys tend to report higher levels of moral disengagement than girls (Sánchez-Jiménez & Muñoz-Fernández, 2021), and that this tendency is associated with an increased likelihood of males perpetrating sexual violence and harassment against females (Navas et al., 2021; Page & Pina, 2018). It is not surprising, then, that the combination of pronounced sexist ideology has been linked to a greater moral disengagement in TFSV scenarios, including DSH (Martínez-Bacaicoa et al., 2024c), as well as with a higher likelihood of engaging in cyberdating abuse (Rollero & De Piccoli, 2020; Sánchez-Hernández et al., 2020 or non-consensual sharing of sexual content (Durán-Guerrero & Sánchez-Jiménez, 2025). However, the specific roles of moral disengagement and sexist beliefs in the actual perpetration of DSH remain underexplored—particularly with regard to potential gender differences in how these mechanisms operate.

### 1.3. Protective Factors Against Perpetration

Understanding the factors that facilitate the use of violence is necessary, but equally important is the exploration of those that serve to discourage it. Within the field of prevention, digital citizenship has gained growing international attention as an educational framework to foster the implementation of internet ethics in society (e.g., Dass & Pramod Kumar, 2024; Richardson et al., 2021). Central to its promotion are online ethical values, as stronger civic and moral attitudes have been associated with lower engagement in unethical online behaviors such as cyberbullying, as well as with higher levels of prosocial online actions, including helping online harassment victims and showing tolerance toward diversity in digital contexts (e.g., Harrison & Polizzi, 2022; Jones & Mitchell, 2016; Kim & Han, 2020; Valdés-Cuervo et al., 2024). However, despite this evidence, the potential protective role of online ethical values in preventing other equally prevalent forms of digital violence, particularly those rooted in gendered power dynamics, has received significantly less attention within this area of research (Henry et al., 2021). Therefore, this study aims to examine the extent to which online ethical values may serve as protective factors against the perpetration of unwanted sexual attention behaviors in digital contexts.

### 1.4. The Present Study

This study seeks to address the pointed gaps in the literature through the following objectives. First, we aim to analyze the prevalence rates of DSH as a function of gender, age, sexual orientation and relationship status. Prior research has consistently shown that DSH and related forms of TFSV are more frequently perpetrated by males than females and by young adults rather than older adults (Martínez-Bacaicoa et al., 2024d; Miguel-Álvaro et al., 2025). However, more research is needed, specifically on DSH perpetration, particularly including other sociodemographic variables such as sexual orientation and the type of relationship with the target. Additionally, we analyze the nature of the relationship between perpetrators and their targets (e.g., current or former partners, people they were getting to know, strangers, or friends), as this aspect has been scarcely considered in previous studies despite its potential relevance to better understand the dynamics in which DSH takes place (Franceschi et al., 2024). The second objective is to identify psychological predictors of DSH perpetration. Previous studies have shown that moral disengagement is a mechanism associated with higher levels of both online and offline sexual harassment (Page & Pina, 2018; Martínez-Bacaicoa et al., 2024c). Likewise, sexist ideology has been linked to an increased likelihood of engaging in various forms of TFSV, including DSH (Martínez-Bacaicoa et al., 2024c). Benevolent sexism may motivate such behaviors as a perceived form of flirting, assuming that the recipient would appreciate it (Oswald et al., 2019), whereas hostile sexism may drive DSH as a way to assert traditional male dominance and sexual entitlement (Tang et al., 2019). Nevertheless, the specific roles of moral disengagement and sexist beliefs in predicting actual DSH perpetration remain underexplored. Therefore, this study examines whether hostile sexism, benevolent sexism, and moral disengagement operate as risk factors for DSH perpetration, while controlling for sociodemographic variables. Regarding protective factors, we analyze whether online ethical values act as a protective variable, as they have been linked to lower perpetration of other online forms of violence, such as cyberbullying, and to higher levels of prosocial online behavior (Jones & Mitchell, 2016; Kim & Han, 2020). However, their role in gendered forms of online sexual violence, such as DSH, remains unknown. We further examine whether the protective effect of online ethical values varies depending on levels of sexism and moral disengagement—that is, whether their buffering role is attenuated under high-risk conditions. Finally, as DSH is a gendered form of violence rooted in broader patterns of social norms and inequality (DeKeseredy et al., 2019), we explore whether the predictive strength of risk (sexism, moral disengagement) and protective (online ethical values) factors differs by gender. Previous studies have reported gender differences in both sexism and moral disengagement in relation to TFSV (Martínez-Bacaicoa et al., 2024c), but little is known about how these mechanisms operate specifically in DSH perpetration. By addressing these objectives, this study seeks to advance current understanding of the factors that contribute to DSH perpetration and to inform the design of prevention strategies that not only focus on victims but also target the mechanisms underlying perpetration. Therefore, based on the existing literature on DSH perpetration, our hypotheses are as follows:

**H1.**  *DSH perpetration will be more prevalent among males than females*.

**H2.**  *DSH perpetration will be more prevalent among adolescents than among young adults*.

**H3.**  *Both benevolent and hostile sexism will be positively associated with the likelihood of DSH perpetration*.

**H4.**  *Moral disengagement will be positively associated with the likelihood of DSH perpetration*.

**H5.**  *Online ethical values will be negatively associated with the likelihood of DSH perpetration*.

**H6.**  *Gender will moderate the effects of ambivalent sexism and moral disengagement on DSH perpetration*.

## 2. Materials and Methods

### 2.1. Participants

1098 Spanish adolescents and young adults aged 13 to 23 years (Mage = 16.07, SDage = 2.38) participated in the study. Among them, 57.7% (n = 634) identified as females, 41% (n = 450) as males, 0.4% (n = 4) as gender diverse, and 0.9% (n = 10) preferred not to specify their gender. Regarding sexual orientation, 83% (n = 911) identified as heterosexual, 2.1% (n = 23) as homosexual, 9.5% (n = 104) as bisexual, 0.6% (n = 7) reported other orientations (e.g., pansexual, asexual), 3.7% (n = 41) were uncertain, and 1.1% (n = 12) preferred not to respond. The majority of the sample was Spanish (84.9%), and the remaining participants were from Latin America (11.5%), Europe (2%), Africa (0.9%), Asia (0.5%) and North America (0.2%). The parental marital status data showed that 67.7% of the participants’ parents were married/cohabiting, 26.1% were divorced/separated, 3.5% were single parents, and 2.5% were widowed. This question was not answered by 0.3% of the participants. In terms of relationship status, 42.6% of the participants were currently in a relationship or had been in one during the past 12 months, while 57.4% indicated that they were single.

### 2.2. Measures

Digital Sexual Harassment Perpetration. We use the Digital Sexual Harassment subscale of the Technology-Facilitated Sexual Violence Scales (Martínez-Bacaicoa et al., 2024d). This scale consisted of five items that assessed the presence of sexual attention behaviors committed through technology and directed at someone who did not wish to receive them (e.g., “You have made sexual comments to someone that you believe they did not want to receive”). Respondents were asked how many times they had engaged in each behavior in the past 12 months through internet platforms (e.g., social media, chats) or mobile phones. This scale has demonstrated good properties, including concurrent and factorial validity, as well as reliability among young adult samples (Martínez-Bacaicoa et al., 2024d). The internal consistency (Cronbach’s alpha) in this study was 0.81, indicating good reliability. Subsequently, to assess the relationship between aggressor and victim, participants who reported having engaged in at least one DSH behavior during the last year were asked to whom their actions had been directed (current partner, former partner, someone they were getting to know, stranger, friend).

Online Ethical Values. The 16-item version of the Online Ethical Values and Behaviors Scale (Alonso-Fernández et al., in press) was used to assess the online ethical values across five dimensions: online respect (e.g., “Respect internet users, even when they share opinions that differ from mine”), online responsibility, prosocial cyberbystander behavior (e.g., “Help other people who are victims of violence on the internet”), tolerance for diversity, and online self-development. Participants rated the importance of each statement to them on a 5-point Likert scale (1 = Not important at all, 5 = Very important). The instrument was previously validated in a sample of Spanish adolescents and young adults, showing good psychometric properties. In the present study, the total score was used as a composite measure of online ethical values, showing good internal consistency (α = 0.90).

Ambivalent Sexism Inventory. This study used the short 12-item version of the Ambivalent Sexism Inventory (ASI; Glick & Fiske, 1996), in its Spanish adaptation by Rodríguez-Castro et al. (2009). The scale consists of 12 items which are statements regarding the relationships between men and women in society: 6 items measure hostile sexism, which reflects overtly negative evaluations of women and considers them inferior to men (e.g., “In general, when a woman is beaten in a fair competition, she complains of discrimination”) and the remaining 6 items measure benevolent sexism, which with a positive affective tone, considers women not inferior to men but different (e.g., “Women are characterized by a purity that few men possess”). Responses are rated on a 5-point Likert scale ranging from 1 (strongly disagree) to 5 (strongly agree), with higher scores indicating higher levels of hostile sexism (Cronbach’s alpha = 0.91) and benevolent sexism (Cronbach’s alpha = 0.78).

Moral disengagement. Moral disengagement was measured using a 9-item scale originally developed by Knauf et al. (2018) to assess moral disengagement mechanisms in school bullying and cyberbullying contexts. In this study, we followed the adaptation framework proposed by Wachs et al. (2022) for hate speech, modifying the items to refer more broadly to online harassment: “When I realize that someone is being harassed (mocked, insulted, humiliated, or discriminated against) on the Internet or social media…”. Nine items were used, which reflect the mechanisms introduced by Bandura et al. (1996), including victim-blaming (e.g., “I figure it’s their own fault”), dehumanizing the victim (e.g., “I assume they don’t deserve anything better”, distorting the negative consequence for the victim (e.g., “I tell myself it’s probably not that bad”), and diffusion of responsibility (e.g., “I think that it’s none of my business”), among others. Participants rated each item on a 5-point Likert scale ranging from 1 (strongly disagree) to 5 (strongly agree), with higher scores indicating greater moral disengagement. This scale has demonstrated satisfactory validity indices among adolescent populations (Wachs et al., 2022), and internal consistency in the present sample was also good (α = 0.88).

Sociodemographic questionnaire. Participants answered questions about their age (calculated using date of birth), gender (girl, boy, trans, non-binary, or prefer not to say), sexual orientation (heterosexual, bisexual, homosexual, other, not sure, or prefer not to say), and country of birth (open-ended question). They also reported their relationship status by indicating whether they were currently or had been in a relationship in the last 12 months, had not been in a relationship in the past year, or whether they had never had a partner.

### 2.3. Procedure

The survey was administered in eight secondary schools of the Community of Madrid (four public, three private and one publicly funded private school) and three Spanish public universities. The research team contacted the schools and universities to obtain institutional approval before data collection. In secondary schools, once authorization was granted, parental consent forms were distributed to students and sent home to be signed for participants under the age of 18. After allowing time to collect the signed consent forms, members of the research team visited the schools to present the study to students who were authorized to participate. These students were then invited to provide their own informed consent, and only those who agreed completed the questionnaire in class. Both consent forms outlined the study’s objectives, clarified the anonymity and voluntariness of participation, and noted that participants could skip any question or withdraw at any time without any consequences. In all secondary schools, data were collected in class during regular school hours, with at least one member of the research team present to administer the questionnaire—either in paper format or via an online platform—answer questions, ensure the appropriateness of the setting, and guarantee privacy and independent completion. Similarly, in universities, institutional approval was obtained, and the study was promoted among students, who could participate voluntarily. University students completed the survey either in class, with members of the research team present, or independently via a Qualtrics link after reading the information sheet. They were also given the option to contact the research team by email or phone at any point—before, during, or after completing the questionnaire—if they had any questions or encountered any issues. To ensure data quality, control procedures were implemented, including the review of response times and the detection of irregular response patterns. The questionnaire took approximately 35 min to complete, as it was part of a broader longitudinal research project conducted by the team on predictors of online violent behavior among youth—particularly online hate speech and the spread of fake news—approved by the Ethics Committee of the Autonomous University of Madrid. Upon completion, all participants—as well as those who were present in the classroom but either lacked parental consent or chose not to participate—received an information sheet listing free mental health and support services available in the Community of Madrid and across Spain, along with the researchers’ contact details.

### 2.4. Statistical Analysis

First, descriptive statistics for the prevalence of DSH perpetration were calculated for each item and for the overall scale. To do so, items were dichotomized (0 = never, 1 = at least once in the past 12 months). Group differences by gender, age, sexual orientation, and relationship status were examined using Pearson’s χ^2^ tests, as all sociodemographic variables were also dichotomized: gender (0 = male, 1 = female), age (0 = 13–17 years, 1 = 18–23 years), sexual orientation (0 = heterosexual, 1 = LGB), and relationship status (0 = single, 1 = in a relationship during the past 12 months). For those who reported engaging in at least one DSH behavior, descriptive statistics were computed, and the nature of the relationship with the target (i.e., current partner, ex-partner, stranger, or friend) was examined. Chi-square tests were used to examine differences in target selection by gender and age. Then, bivariate correlations among all study variables were computed using Pearson’s r for continuous–continuous pairs, the Phi coefficient for binary–binary pairs, and point-biserial correlations for binary–continuous combinations.

As the aim of the study was to examine differences between individuals who had and had not perpetrated DSH, rather than treating the outcome as continuous, we conducted hierarchical binomial logistic regression analyses. In Step 1, we tested the predictive role of online ethical values, ambivalent sexism, and moral disengagement. Gender, age, sexual orientation, and relationship status were entered as control variables. In Step 2, we tested interaction effects between the main predictors and gender to explore potential moderating effects. All continuous predictors were mean-centered, and sociodemographic variables were dummy-coded to facilitate interpretation. Descriptive analyses were performed using SPSS version 28.0, and correlation and binomial regression analyses were conducted using RStudio version 2025.5.1.513.

To ensure the adequacy of the binomial regression model, we tested the assumptions of linearity, no multicollinearity, independence of residuals, and appropriate dispersion relative to the mean for all predictor variables (Pardo & Ruiz, 2012). No violations were detected. Approximately 9.1% of the data was missing, reducing the sample size from 1098 to 998 complete cases for the regression analysis. To address this, we first visually inspected the missing data patterns and found no evident structure. To formally assess the missing data mechanism, we conducted the non-parametric version of the TestMCARNormality from the MissMech package version 1.0.4 (Jamshidian et al., 2014), suitable for categorical variables. Results indicated no significant evidence to reject the assumption that the data were Missing Completely At Random (MCAR; *p* = 0.13). Therefore, listwise deletion was applied in the logistic regression analysis, as the data were deemed not to introduce systematic bias.

Finally, considering the asymmetry of the phenomenon under study, a threshold adjustment was applied to improve the model’s predictive performance. We examined the Receiver Operating Characteristic (ROC) curve to improve both sensitivity and specificity. Two criteria were compared: (1) a threshold based on the minority class proportion (cutoff = 0.125), which yielded an accuracy of 0.60, sensitivity = 0.60, specificity = 0.72, and balanced accuracy = 0.71; and (2) a threshold based on the optimal point of the ROC curve (cutoff = 0.118), which improved performance to accuracy = 0.69, sensitivity = 0.68, specificity = 0.78, and balanced accuracy = 0.73. Given that identifying perpetrator profiles was a priority, the ROC-based threshold was selected.

## 3. Results

### 3.1. Prevalence of DSH Perpetration and Gender, Age, Sexual Orientation and Relationship Status Differences

We examined the prevalence of having engaged in DSH behaviors by dichotomizing item responses (0 = never, 1 = at least once in the past year). Among the 1098 participants who completed the DSH perpetration scale, 13.4% reported having engaged in at least one such behavior in the past 12 months. Prevalence rates for individual items ranged from 2.5% (sending sexual content that was unwanted by the recipient) to 8.3% (asking unwanted sexual questions). Table 1 presents prevalence rates and differences by gender, age, sexual orientation, and relationship status.

Gender differences were observed across all items and in the total DSH score, with male participants consistently reporting higher rates of perpetration than female participants. Specifically, 21.1% of males reported at least one form of DSH, compared to 7.9% of females (*p* < 0.001). Age differences were also significant, with adolescents aged 13–17 reporting higher overall DSH rates (16.1%) than those aged 18–23 (6.5%, all *p* < 0.001). No significant differences in DSH perpetration were found based on sexual orientation or relationship status.

Descriptive statistics were also analyzed for participants who reported having perpetrated at least one form of DSH in the past year. Among the perpetrators, most were male participants (65.5%), and the majority (86.4%) were underage. Regarding sexual orientation, most male participants identified as heterosexual (89.5%), while a greater proportion of female participants identified as bisexual (26%). Notably, around 8% of both male and female participants who perpetrate DSH reported being unsure about their sexual orientation. Approximately half of the perpetrators (51%) were in a romantic relationship or had been in one within the past year.

Finally, exploratory analyses of the relationship to the target revealed that DSH behaviors were most often directed toward someone the perpetrator was getting to know romantically or sexually (32.4%), followed by strangers (21.9%), current partners (19%), friends (17.1%) and former partners (9.5%). Gender and age differences in target selection were also observed. Compared to female participants, male participants were more likely to direct their DSH behavior toward strangers (29% vs. 12.2%, *p* < 0.05), while female participants more often targeted current partners (31.7% vs. 11.3%; *p* < 0.01) and friends (26.8% vs. 11.3%; *p* < 0.05). Regarding age, young adults aged 18–23 were significantly more likely to report targeting friends (45% vs. 10.6%, *p* < 0.001).

### 3.2. Relationship Between Digital Sexual Harassment Perpetration and Online Ethical Values, Ambivalent Sexism and Moral Disengagement

Table 2 displays means, standard deviations, and correlations among all the study variables. Regarding gender differences in mean scores on the predictor variables, higher levels of online ethical values were observed among female participants, whereas male participants reported higher levels of both hostile and benevolent sexism, as well as moral disengagement. In terms of associations between DSH perpetration and the examined risk and protective factors, DSH was significantly and negatively correlated with online ethical values (−0.25, *p* < 0.001), suggesting that individuals with lower ethical values in online contexts were more likely to engage in DSH. In contrast, positive correlations were found between DSH and benevolent sexism (0.20), hostile sexism (0.24) and moral disengagement (0.20, all *p* < 0.001), indicating that higher levels of sexist attitudes and justification of online harassment were associated with greater DSH perpetration. Among the sociodemographic variables, gender and age were negatively correlated with DSH, reflecting higher DSH perpetration among male and younger participants. No significant associations were found between DSH and sexual orientation or relationship status.

### 3.3. Risk and Protective Predictors of Digital Sexual Harassment Perpetration

A hierarchical binomial logistic regression was conducted to examine the predictors of DSH perpetration. In Step 1, the model included online ethical values, benevolent and hostile sexism, and moral disengagement, while controlling for gender, sexual orientation, and relationship status. Age was excluded from the final model, as it was not significant and its removal improved the model fit. As shown in Table 3, higher levels of values significantly reduced the likelihood of engaging in DSH, with each unit increase in ethical values associated with a 47.5% decrease in the odds of perpetration (ExpB = 0.525, *p* < 0.001), while higher levels of benevolent sexism were associated with a 42.5% increase in the odds of DSH (ExpB = 1.425, *p* < 0.05). Hostile sexism and moral disengagement showed positive trends but did not reach statistical significance in this step.

In the second step, interactions were included between the predictors of DSH—both risk and protective factors—as well as their interactions with gender. The final model retained only the three significant interactions, leading to an increase in explained variance from 17.1% to 19.4% (ΔR^2^ = 0.054, Nagelkerke R^2^ = 0.194). The remaining interaction terms were excluded to maintain parsimony and facilitate the interpretation of the model’s parameters. Regarding the main effects of the predictors, online ethical values remained a significant protective factor, and benevolent sexism continued to be a significant predictor. Moral disengagement became a significant risk factor in this step, doubling the odds of DSH for each unit increase (ExpB = 2.05, *p* < 0.01). Among the sociodemographic controls, identifying as LGB (lesbian, gay, bisexual) and being in a romantic relationship were both significantly associated with higher odds of perpetration (β = 1.018 and β = 0.460, respectively).

**Figure 1:**
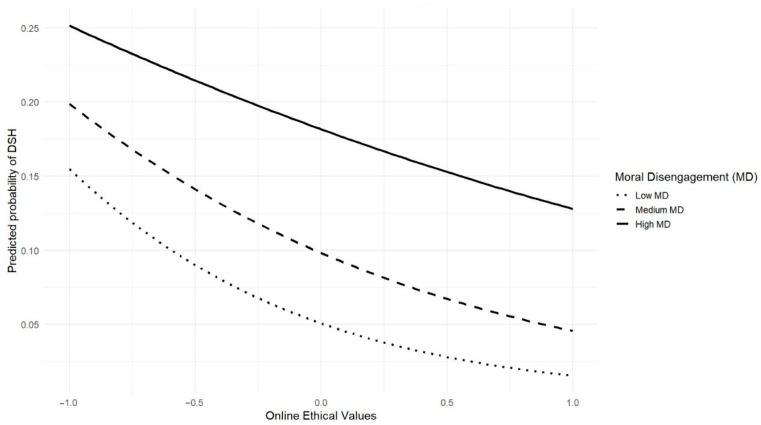
Moderating Role of Moral Disengagement in the Relationship Between Online Ethical Values and Probability of DSH Perpetration.

Regarding the moderating role of the principal variables, Figure 1 shows the interaction between online ethical values and moral disengagement (β = 0.409, SE = 0.208, OR = 1.51, 95% CI [1.00, 2.26], *p* < 0.05). The interaction revealed that the protective effect of ethical values against the perpetration of DSH was weaker at higher levels of moral disengagement. In other words, although higher online ethical values consistently reduce the likelihood of perpetration, individuals with elevated moral disengagement still present a greater overall probability of engaging in DSH compared to those with lower moral disengagement.

**Figure 2:**
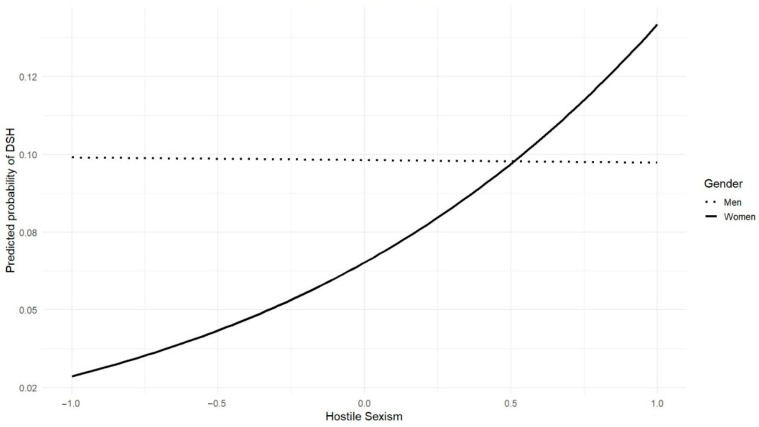
Moderating Role of Gender in the Relationship Between Hostile Sexism and Probability of DSH Perpetration.

**Figure 3:**
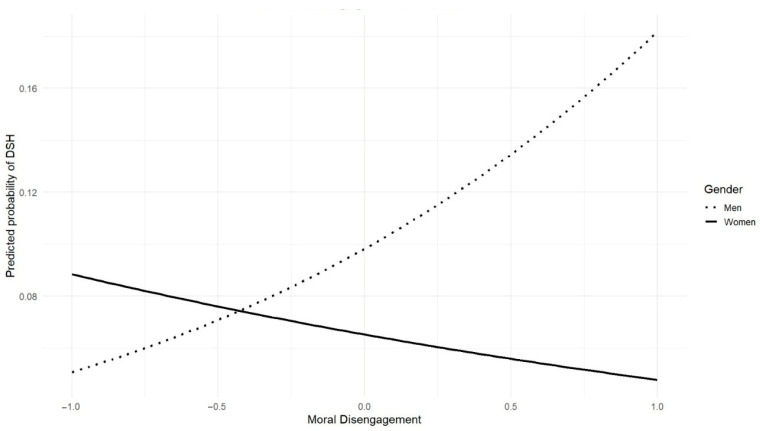
Moderating Role of Gender in the Relationship Between Moral Disengagement and Probability of DSH Perpetration.

Additionally, when examining gender differences in the predictors of DSH, hostile sexism was a significant predictor only for female participants, nearly doubling their odds of perpetration (ExpB = 2.39, *p* < 0.01; see Figure 2), whereas moral disengagement was significant only for male participants (β = −1.042, *p* < 0.05; see Figure 3). The constant remained significant (β = −2.222, *p* < 0.001), reflecting low baseline odds for the reference group (heterosexual, single males with average scores on online ethical values, moral disengagement, and sexism).

## 4. Discussion

DSH perpetration among youth is a concerning issue that requires greater attention in academic research. The aim of this study was to address existing gaps in the literature and examine the prevalence of DSH perpetration according to gender, age, sexual orientation, and relationship status. In addition, the study explored potential risk factors (hostile sexism, benevolent sexism, and moral disengagement) and protective factors (online ethical values), as well as their differences by gender. The results revealed that DSH is a prevalent issue, with 13.4% of participants reporting having engaged in DSH during the past 12 months. When examining specific behaviors, the least reported forms of DSH involved sending unsolicited sexual images (2.5%) and pressuring others to send sexual images (3%), while unwanted sexual comments or questions were reported more frequently (7–8%), suggesting that such behaviors may be more socially tolerated or less clearly identified as harassment by youth. Higher prevalence rates of perpetration have been found in a recent study among Spanish adolescents (Durán-Guerrero et al., 2025), may be due to the fact that their study includes broader categories of online sexual behavior, while similar rates were found in previous studies when specifically addressing unwanted sexual attention (Ybarra & Petras, 2021). This suggests that differences across studies may depend on the operationalization of DSH.

As expected in our hypothesis, gender and age differences emerged. Male participants reported more than twice the rates of perpetration compared with female participants (21.1% vs. 7.9%), and adolescents showed a higher prevalence than young adults (16.1% vs. 6.5%). These results are in line with the gendered pattern found in previous studies, where males are more frequently identified as perpetrators of DSH (Durán-Guerrero et al., 2025; Gámez-Guadix et al., 2023; Ramljak et al., 2025) and females as the most frequent victims (Buchanan & Mahoney, 2022; Powell & Henry, 2019; Martínez-Bacaicoa et al., 2024b; Ybarra & Petras, 2021). With regard to the age differences, higher rates among adolescents may reflect developmental factors, as this stage is characterized by sexual curiosity and identity exploration, often accompanied by limited awareness of risks and consequences (Barrense-Dias et al., 2020). This exploration occurs within a context where traditional gender norms establish that male sexual desire is natural, while female sexuality remains subordinate to that desire. Sometimes, these ideas can lead to the normalization of behaviors such as persuasion or sending unsolicited sexual content, which makes them less clearly identifiable as harassment (Hunehäll-Berndtsson & Odenbring, 2020; Mikorski & Szymanski, 2017 Thomas, 2018).

Although the exploratory analyses revealed no significant differences in DSH perpetration based on sexual orientation or relationship status, identifying the targets of such violence may still provide valuable insights. The most prevalent target was someone they were getting to know, followed by strangers, current partners, friends, and former partners, with differences according to gender and age. Female participants and young adults engage in DSH in closer relational contexts—such as current partners or friends—while male participants and adolescents tend to take more risks and direct these behaviors outside their immediate circles. These findings are consistent with previous evidence showing that DSH is often motivated by partner seeking or personal or sexual gratification (Karasavva et al., 2023; Oswald et al., 2019), and that while females often receive unsolicited sexual images from unknown males, their own sexting practices are more often engaged in romantic contexts, sometimes framed as a relationship requirement or part of “empowered” female sexuality (Mishna et al., 2021; Thorburn et al., 2021).

In relation to the second objective of the study—to analyze risk and protective factors of DSH perpetration while controlling for sociodemographic variables—the results showed that, although gender and age correlated with higher perpetration, their effects disappeared when other predictor variables were included in the regression models, suggesting that they are not determining factors in themselves, but rather depend on the surrounding sociocultural contexts of gender and sexual socialization (Hardt et al., 2022; Brown et al., 2020; Bianchi et al., 2017). Notably, identifying as LGB and being in a romantic relationship were associated with increased odds of perpetration. These findings may reflect specific contexts where boundaries around consent are more easily blurred, such as dating apps, used for sexual exploration by LGB youth (Sharabi et al., 2022; Wu & Ward, 2018) or within romantic relationships, where adolescents may perceive sexual requests as a relational expectation (Hertzog & Rowley, 2014; Lewis et al., 2021).

Regarding risk factors measured in our study, our hypothesis was only partially supported. While both hostile and benevolent sexism correlated with DSH perpetration, only benevolent sexism remained a significant predictor in the regression model, and this effect did not differ by gender. This finding is consistent with recent studies showing that behaviors such as sending unsolicited sexual content or making body-related compliments may be motivated by sexual excitement or perceived as something the recipient would appreciate, yet they are rooted in ambivalent sexist attitudes and serve to reinforce gendered power dynamics (Gutierrez & Leaper, 2024; Oswald et al., 2019; Rodríguez-Castro et al., 2025).

Moral disengagement also emerged as a significant predictor once interactions were included, reinforcing its role as a facilitator of DSH. This result aligns with recent studies highlighting its relevance in TFSV perpetration, including DSH (Martínez-Bacaicoa et al., 2024c; Ojeda et al., 2024). Given that moral disengagement allows perpetrators to justify, minimize, or emotionally distance themselves from their actions and the potential harm caused, it seems understandable that it would predict a higher likelihood of committing DSH. This mechanism is particularly effective in digital contexts, characterized by anonymity, lack of authority, and difficulty in establishing clear legal boundaries, which encourages the diffusion of responsibility and fosters TFSV behaviors among individuals, especially those with sexist attitudes (Henry & Powell, 2015; Suler, 2004; Zhong et al., 2020).

As partially postulated in our hypothesis, gender moderated the effects of hostile sexism and moral disengagement on DSH perpetration. Specifically, high levels of hostile sexism predicted perpetration only among female participants, whereas high levels of moral disengagement predicted perpetration only among male participants. Different pathways may account for this pattern, reflecting distinct socialization processes. Men are often socialized into traditional masculinity and sexual stereotypes, which normalize insistent or dominant sexual behavior and facilitate moral disengagement as a legitimizing mechanism for unwanted sexual attention without explicitly relying on sexist attitudes (Khera et al., 2022; Thorburn et al., 2021). In contrast, women, typically socialized to be less sexual and non-aggressive, may require more internalized hostile sexist beliefs to justify engaging in such behaviors. This interpretation is consistent with evidence showing that females’ justification of TFSV is influenced by sexist beliefs (Martínez-Bacaicoa et al., 2024c) and that tolerating sexism can increase their acceptance of sexual harassment (Mallett et al., 2019).

It should be noted that the predominantly Spanish demographic in this study may have influenced these results. In Hispanic cultures, traditional gender ideologies have historically played a strong role in shaping interpersonal and sexual relationships, with both girls and boys showing higher tolerance toward benevolent sexism and romanticized ideals of love (Donoso-Vázquez et al., 2018; Estébanez, 2018). These cultural patterns may contribute to the normalization or minimization of certain DSH behaviors, especially those that could be perceived as expressions of flirtation rather than as forms of violence. However, in recent years, Spanish society has undergone significant progress toward gender equality, aligning more closely with other Western European countries. This evolving context may have also influenced the way young people interpret and report some DSH behaviors.

Lastly, a novel contribution of this study was the identification of online ethical values as a protective factor, consistently associated with lower odds of perpetration. While previous research has linked prosocial attitudes and ethical values to reduced involvement in cyberbullying (Jones & Mitchell, 2016; Nickerson et al., 2022), no clear empirical evidence has yet been provided regarding their preventive role in gendered online forms of violence such as DSH. Our interaction analyses further revealed that when online ethical values were low, the likelihood of perpetration remained high regardless of moral disengagement levels. In contrast, high levels of online ethical values substantially reduced the probability of perpetration, except in cases of very high moral disengagement, where the risk, although attenuated, persisted. This pattern is consistent with the notion that moral disengagement undermines self-regulatory processes, but its impact is considerably weaker when individuals strongly endorse online ethical values. These findings reinforce the protective role of online ethical values against TFSV and highlight their importance as a focus of future research.

### 4.1. Implications for Practice and Research

Although Spain and Western societies have made significant progress toward gender equality and the rights of sexual minorities (Moya & Moya-Garófano, 2021; Organization for Economic Co-Operation and Development [OECD], 2020), specific prevention programs addressing TFSV remain scarce in Spain. The present study has several practical implications in this regard. First, the prevalence rates observed confirm that DSH is not an isolated phenomenon among youth, even though self-reported perpetration is lower than victimization rates documented in prior studies (e.g., Martínez-Bacaicoa et al., 2024b). This discrepancy suggests that youth may have difficulties in detecting or acknowledging some DSH behaviors. Notably, most participants did not report engaging in pressured sexting, yet other forms of DSH were more frequent, where the boundaries between consent and coercion may be poorly understood, socially tolerated, or misinterpreted as forms of flirting or sexual exploration. These findings emphasize the need for early prevention strategies that help adolescents better understand boundaries and the importance of consent, explicitly addressing blurred lines between sexual interest, flirting, and coercion, helping youth differentiate DSH from consensual sexting, which can constitute a healthy expression of adolescent sexuality (Bianchi et al., 2017; Mishna et al., 2021).

It is essential to acknowledge that DSH has deep social roots; therefore, prevention should not fall solely on potential victims or focus only on safe online practices. Instead, prevention efforts must target the variables that act as risk or protective factors. In this regard, the identification of benevolent sexism and moral disengagement as significant risk factors, and online ethical values as a consistent protective factor, underscores the importance of implementing comprehensive digital citizenship education programs with a gender perspective that explicitly addresses respect and consent in online interactions and the recognition of DSH behaviors. Beyond theoretical knowledge, school-based interventions should include online ethical dilemmas and experiential activities that reduce moral disengagement, challenge traditional gender norms and romantic myths, and promote critical reflection about responsibility and emotional boundaries in digital interactions.

In addition, the moderating role of gender further indicates the need to move beyond a male-centered view of perpetration, as our results suggest that both male and female participants may engage in DSH, but through different pathways: for males, moral disengagement appears to be the key mechanism, reinforced by norms of traditional masculinity that normalize persistent pursuit, while for females, hostile sexist beliefs seem to play a more relevant role in justifying such behaviors. These different mechanisms highlight the importance of prevention strategies that address the root of the problem by explicitly tackling gendered socialization processes and challenging harmful stereotypes in both groups. Through sexist ideology, public discourse still tends to place responsibility on victims—especially women—by framing them as careless or provocative (Zhong et al., 2020), reflecting traditional sexual scripts in which women are expected to say “no” while men are allowed to ignore their refusal (Estébanez, 2018). Conversely, men are perceived as having an inherent and uncontrollable sexual desire, and some women may interpret their own DSH behaviors toward men as flirtatious rather than inappropriate, while male victims may feel uncertain or ashamed about their reactions, as these experiences conflict with gender expectations that assume they should enjoy such attention (Erentzen et al., 2023).

Finally, the higher odds of perpetration found among LGB youth and those in relationships highlight the importance of tailored interventions in these contexts, where norms of consent may be less clearly defined. However, it should be noted that the higher proportion of heterosexual participants compared to LGB individuals in this study may have influenced the observed results regarding perpetration odds. Future research is therefore needed, particularly relevant for lesbian and bisexual female participants and for young people with gender diverse identities, whose experiences remain underexplored in the literature.

### 4.2. Limitations and Future Lines of Research

This study presents some limitations that should be taken into account. Firstly, although anonymity and confidentiality were ensured during data collection to encourage honest reporting, the self-report nature of the data may introduce a degree of bias (e.g., social desirability). Moreover, the figures may not be entirely accurate, as some participants may have normalized DSH behaviors or may not have been aware that their actions were perceived as unwanted by the person to whom it was directed, and therefore did not report it in this study. Conversely, some participants—mainly females—may show greater self-awareness or empathy when assessing whether their behavior was unwanted (Alonso-Fernández et al., in press), which could also influence higher reporting rates. Future studies should take into account this potential bias in perception and awareness when analyzing DSH prevalence. In this regard, qualitative studies could explore the nuances and perceptions that male and female participants have regarding what constitutes DSH. Secondly, the young adult sample (18–23) was predominantly comprised of female participants, who are known to perpetrate to a lesser extent (Martínez-Bacaicoa et al., 2024d). This imbalance in the sample may have influenced the lower perpetration rates in this age group, contributing to larger prevalence differences with other age groups. In addition, the number of LGB participants was relatively small compared to heterosexual participants, which may have biased the results regarding the higher odds of perpetration among LGB youth and reduced the statistical power of the analyses. Future research should include more balanced samples across all age ranges, as well as individuals with non-normative gender identities and sexual orientations. Thirdly, the adolescent sample consisted mainly of Spanish participants, and the percentage of perpetrators in this group was very small, which likely contributed to the small effect sizes observed. Therefore, caution should be exercised when generalizing these findings to other Hispanic or non-Western contexts, where gender norms and digital behaviors may differ substantially. Future studies should be conducted with larger sample sizes and with participants from different countries. In this regard, it is also important to consider that both gender roles and perceptions of what constitutes DSH are influenced by cultural norms; therefore, in different cultural contexts, the interpretation and prevalence of these behaviors may vary. Finally, the cross-sectional nature of this study prevents the establishment of causal relationships between the variables analyzed and DSH perpetration. Future research should employ longitudinal designs to track changes in DSH behavior over time and to better understand the temporal and causal relationships between risk—ambivalent sexism and moral disengagement—and protective factors—online ethical values.

## 5. Conclusions

This study contributes to a more comprehensive understanding of DSH by focusing on perpetration, which is a dimension often overlooked in favor of victimization. With 13.4% of youth—mostly males—reporting perpetration in the past year, our findings underscore the need to identify and prevent risk factors among potential perpetrators. Recognizing the mechanisms that underlie perpetration is essential to designing prevention strategies that move beyond victim support and address the roots of DSH. Ambivalent sexism and moral disengagement play a key role in enabling DSH by allowing perpetrators to justify, minimize, or emotionally distance themselves from their actions, with gendered dynamics shaping these processes. Importantly, this is the first study to demonstrate the protective role of online ethical values against DSH perpetration, opening new avenues for research on digital citizenship from a gender- and intersectionality-informed perspective.

## Figures and Tables

**Table 1:** Prevalence of DSH perpetration and Differences by Gender, Age, Sexual Orientation and Relationship Status.

	Total	Gender			Age			Sexual Orientation			Relationship Status		
		Males	Females	χ^2^	13–17	18–23	χ^2^	Heterosexual	LGB	χ^2^	Single	Relation	χ^2^
		n = 463	n = 636		n = 787	n = 310		n = 909	n = 136		n = 629	n = 466	
You have made sexual comments to someone that you believe they did not want to receive.	6.9%	12.4%	3%	36.48 ***	8.6%	2.6%	12.67 ***	6.9%	5.2%	0.54	6.5%	7.5%	0.41
You have asked someone sexual questions that you believe they did not want to answer.	8.3%	14%	4.1%	34.22 ***	9.7%	4.8%	6.79 **	8.1%	7.5%	0.07	7.6%	9.2%	0.90
You have pressured someone to send you sexual photos or videos that you believe they did not want to send.	2.6%	4.5%	1.3%	10.64 ***	3.4%	0.6%	6.72 **	2.7%	1.5%	0.73	2.1%	3.4%	1.96
You have pressured someone to answer sexual questions that you believe they did not want to respond to.	3.6%	6.4%	1.6%	17.98 ***	4.6%	1.3%	6.83 **	3.7%	1.5%	1.76	2.7%	4.9%	3.79 *
You have sent someone sexual content (photos or videos) of yourself that you believe they did not want to receive.	2.5%	3.8%	1.4%	6.25 **	3.2%	0.6%	5.94 *	2.5%	1.5%	0.53	2.1%	3%	0.98
Digital Sexual Harassment Total	13.4%	21.1%	7.9%	39.73 ***	16.1%	6.5%	17.98 ***	13%	13.4%	0.02	11.9%	15.5%	2.87

Note. Prevalence refers to respondents who reported having been perpetrators of DSH at least once in the last 12 months. *** *p* < 0.001, ** *p* < 0.010, * *p* < 0.05.

**Table 2:** Means, Standard Deviations, and Correlation Matrix Between Variables.

Variable	1	2	3	4	5	6	7	8	9
1. Digital sexual harassment									
2. Online Ethical Values	−0.25 ***								
3. Benevolent Sexism	0.20 ***	−0.28 ***							
4. Hostile Sexism	0.24 ***	−0.45 ***	0.61 ***						
5. Moral Disengagement	0.20 ***	−0.42 ***	0.37 ***	0.50 ***					
6. Gender	−0.19 ***	0.38 ***	−0.29 ***	−0.58 ***	−0.36 ***				
7. Relationship Status	0.05	0.01	0.01	−0.07 *	−0.04	0.14 ***			
8. Sexual Orientation	0.01	0.13 ***	−0.23 ***	−0.21 ***	−0.11 ***	0.16 ***	0.09 **		
9. Age	−0.12 ***	0.28 ***	−0.33 ***	−0.39 ***	−0.28 ***	0.37 ***	0.14 ***	0.26 ***	
Males Mean (SD)	0.61 (1.80)	3.83 (0.72)	2.49 (0.87)	2.68 (1.04)	0.90 (0.65)	-	0.6 (0.23)	0.34 (0.47)	15.17 (1.74)
Females Mean (SD)	0.15 (0.69)	4.33 (0.49)	1.99 (0.72)	1.52 (0.58)	0.48 (0.45)	-	0.17 (0.37)	0.49 (0.5)	16.71 (2.43)
Total Mean (SD)	0.13 (0.34)	4.12 (0.64)	2.19 (0.82)	1.99 (0.98)	0.65 (0.58)	0.58 (0.49)	0.13 (0.34)	0.43 (0.50)	16.07 (2.38)

Note. Pearson’s *r*, Phi, and point-biserial correlations are reported, depending on variable type. Mean (SD) values are presented for the total sample and separately by gender. Gender (0 = male; 1 = female); sexual orientation (0 = heterosexual; 1 = LGB); relationship status (0 = single; 1 = in a relationship). *** *p* < 0.001, ** *p* < 0.010, * *p* < 0.05.

**Table 3:** Binomial Logistic Regression Models.

	Digital Sexual Harassment Perpetration					
	Step 1			Step 2		
	*β*	*SE*	Exp(B) [CI].	*β*	SE	Exp(B) [CI].
Online Ethical Values	−0.645 ***	0.160	0.525 [0.38–0.72]	−0.824 ***	0.183	0.439 [0.31–0.63]
Benevolent Sexism	0.354 *	0.146	1.425 [1.07–1.89]	0.303 *	0.147	1.353 [1.01–1.81]
Hostile Sexism	0.188	0.140	1.207 [0.92–1.59]	−0.009	0.153	0.991 [0.73–1.34]
Moral Disengagement	0.252	0.184	1.287 [0.90–1.84]	0.716 **	0.251	2.05 [1.25–3.35]
Gender	−0.501	0.262	0.606 [0.36–1.01]	−0.429	0.259	0.651 [0.39–1.08]
Relationship Status	0.398	0.207	1.489 [0.99–2.23]	0.460 *	0.209	1.583 [1.05–2.38]
Sexual orientation	0.916 **	0.314	2.499 [1.35–4.63]	1.018 **	0.322	2.767 [1.47–5.20]
Online Ethical Values × Moral Disengagement				0.409 *	0.207	1.505 [1.00–2.26]
Hostile Sexism × Gender				0.871 **	0.285	2.39 [1.37–4.18]
Moral Disengagement × Gender				−1.042 *	0.450	0.353 [0.15–0.85]
Constant	−2.253 ***	0.203	0.105	−2.222 ***	0.212	0.108
R^2Nagelkerke^			0.171			0.194
−2 Log likelihood			658.01			644.658
ΔR^2^			0.171			0.054

Note. 0 = male 1 = female; 0 = heterosexual 1 = LGB; 0 = single 1 = in a relationship. *** *p* < 0.001, ** *p* < 0.010, * *p* < 0.05.

## Data Availability

The data presented in this study are available on request from the corresponding author. The data are not publicly available due to ethical restrictions related to the protection of participant privacy.
